# Developmental progression of bottle-feeding in the first year of life

**DOI:** 10.1016/j.earlhumdev.2026.106507

**Published:** 2026-02-04

**Authors:** Alaina Martens, Jessica Davidson, Katharine Radville, Natalie Peterman, Hayden Kamiya, Kristen Allison, Emily Zimmerman

**Affiliations:** a Department of Communication Sciences and Disorders, Northeastern University, Boston, MA, USA

**Keywords:** Full-term, Bottle, Feeding, Infants

## Abstract

Nutritive sucking (NS), the primary nutritional intake mechanism for infants, represents the cornerstone of early feeding development. NS depends on the integration of efficiency, safety, and coordination to ensure adequate growth and development. Prior work has examined feeding skills at single timepoints. However, information on feeding proficiency and effectiveness across the course of development is limited. Further, despite the importance of nutrition as the main purpose for infant feeding, few clinical instruments exist for quantifying infant feeding physiology. Twenty-seven mother-infant dyads participated in this prospective, longitudinal study with repeated measures at three, six, and nine months. Oral feeding skills were evaluated using the Oral Feeding Skills (OFS) Scale to gather quantitative measurements of infants' feeding abilities [1]. The scale was completed while caregivers bottle-feed their infant. The initial volume of milk offered increased significantly with age. Transfer rate was significantly faster at nine months as compared to at three months. There were no significant effects of age on OFS proficiency or overall transfer volume at any timepoint. These findings provide insights into caregiver practices and infant maturation of bottle feeding. They suggest that while the mechanics of feeding (speed, volume) change with development, fundamental feeding behavior and appetite regulation remain stable.

## 1. Introduction

Nutritive sucking (NS), the primary nutritional intake mechanism for infants, represents the cornerstone of early feeding development. Successful NS depends on the integration of efficiency, safety, and coordination to ensure adequate growth and development. Coordination of suck-swallow-breathe patterning allows for rapid extraction of liquid from the nipple (breast or bottle) and prompt swallow of subsequent boluses with associated disruption in respiratory patterning. This patterning allows for ingestion of nutrients early in life to promote overall growth and maturation [2].

There are two key aspects of NS: expression/compression (tongue pressing liquid out against the hard palate) and suction (negative pressure to draw liquid in). Expression develops first and is present even in very preterm infants, though swallowing remains immature [3]. Suction requires coordinated jaw, soft palate, and lip movements [4]. These create negative pressure inside the oral cavity to maintain the nipple in position and to extract milk [5]. Skills progress from weak suction to strong and from sporadic expression to rhythmic [4]. Prior studies have examined ways in which feeding skills at single timepoints may present themselves as mature or immature. However, information on feeding proficiency and effectiveness across the course of development is limited. Despite the importance of nutrition as the main purpose for infant feeding, few clinical instruments exist for quantifying infant feeding physiology.

Two frequently utilized assessments for evaluating oral feeding in infants are the Early Feeding Skills (EFS) and the Neonatal Eating Assessment Tool (NeoEAT), both of which can be administered by clinicians and parents. The EFS is used for infants born prematurely and for full-term infants through six months of age. It assesses an infant's oral feeding readiness, tracks their feeding development, and evaluates the effectiveness of feeding interventions. It includes a detailed scoring system based on observing the infant's entire feeding session and noting their sustained skills or difficulties. However, the five subscales (Respiratory Regulation, Oral-Motor Function, Swallowing Coordination, Engagement, and Physiological Stability During Feeding) are judged on a 3-point scale for consistency of skill, which does not allow for understanding of ability or adaptability to complete a feed or efficiency of the feed over time. Another commonly utilized assessment tool is the Neo-EAT, which examines problematic feeding from birth to 7 months [6,7]. However, the subscales (Gastrointestinal Tract Function, Infant Regulation, Energy & Physiologic Stability, Sensory Responsiveness, and Feeding Flexibility) place emphasis on the infant's state and physiology before, during, and after feeding.

While these two metrics are excellent at identifying infant readiness for oral feeding and physiological stability, neither of them takes into consideration the volume taken or time spent feeding during a feed, two values that highlight an infant's overall energy expenditure and ability to maintain adequate growth and nutrition orally. Further limitations of a parent-report-based measure include variations in health literacy and noted observations of an infant. Research also shows that parents are more likely to report feeding concerns when there are concerns for weight or growth [8] or when it is a first child [9–11] or based on birth order [9].

The Oral Feeding Skills (OFS) Scale evaluates proficiency and endurance during infant bottle-feeding [1]. This tool was originally developed to objectively and quantitatively assess preterm infants' (26–36 weeks' gestation age) feeding skills at their first oral feeding. Specifically, the scale integrates proficiency, defined as the proportion of prescribed volume consumed in the first 5 min of feeding, with rate of transfer, the average volume transferred across the entire feeding session. These two metrics were used to classify infants into four levels of feeding maturity, ranging from the most immature (low proficiency and low transfer rate) to the most advanced (high proficiency and high transfer rate). Lau and Smith (2011) found that more mature OFS levels were associated with greater overall transfer and shorter feeding duration, more advanced gestational age, and fewer days from the start of oral feeding to independent oral feeding. McGrattan et al. (2023) investigated normative values for healthy, full-term infants using the OFS scale, assessing feeding performance at two feeding sessions within seven days of each other (average infant age 4 ± 2 months) [12]. Their findings revealed that infants consumed milk at an average rate of 7 ± 3 mL/min with a modified proficiency of 50 ± 21%. They also found that infants showed a high variability across the two feeding sessions, with a 40% change in the amount of milk consumed, a 40% change in modified proficiency, and a 54% change in rate of transfer. This initial work provides foundational data for understanding normative feeding metrics in this population [12] but leaves a paucity of data on how these metrics change over time as infants age.

Despite emerging studies in the literature, there remains a lack of comprehensive data on bottle feeding performance across the first year of life in typically developing infants. This gap complicates efforts to establish benchmarks for infants with feeding difficulties and to identify target metrics for therapeutic interventions. Longitudinal studies assessing infant feeding performance at multiple timepoints are essential to understanding developmental trajectories and informing clinical practices. Therefore, the purpose of this study was to examine changes in infant bottle-feeding outcomes across three timepoints (3, 6, and 9 months) in the first year of life using the OFS scale. We hypothesized that as infants aged, they would increase their feeding proficiency, milk transfer rate, and overall transfer volume.

## 2. Methods

### 2.1. Design and participants

his study included a subset of 27 mother-infant dyads enrolled in a larger, ongoing longitudinal study of sucking and motor speech outcomes. Participants were recruited via study flyers, social media posts, and word of mouth. Informed consent was obtained from caregivers for infant participation, and the study protocol was approved by the Northeastern University institutional review board at our institution, IRB #21–08–19. Infants with congenital or chromosomal anomalies or neurological disorders were excluded from the larger study. This smaller analysis included infants who had completed study visits at three, six, and nine months of age. Participants were included in this smaller analysis if they were full-term, had prior bottle-feeding experience, and had OFS scale data at each timepoint. Table 1 provides infant demographic information, and Table 2 provides caregiver demographic information.

### 2.2. Study procedure

The study procedure included an intake form, which provided demographic, feeding history, and bottle-feeding frequency information. Approximately 1 h before the infant's expected feeding time, a member of the research team arrived at the family's home to conduct the study. During the study session, researchers observed the infant bottle-feeding and completed the OFS Scale. Study procedures were the same at the study's 3, 6, and 9 month timepoints.

### 2.3. Oral feeding skills scale

Oral feeding skills were evaluated using the OFS Scale [1], which provides a quantitative assessment of an infant's feeding abilities. The scale was completed while observing caregivers bottle-feed their infant. Caregivers were instructed to feed their infant as they normally would and use their home bottle system. Testing infants in their natural feeding context with familiar equipment and caregivers provides the most accurate assessment of feeding skills, as performance can be significantly influenced by environmental comfort, caregiver familiarity, and bottle system characteristics. This approach minimizes stress-related artifacts and captures the infant's typical feeding patterns. During the observation, the researcher recorded the total volume offered in the bottle, the volume consumed during the first 5 min of feed, the total volume consumed, and the total feeding duration. The following outcomes were calculated: initial volume (mL); transfer volume, (% volume taken/total volume prescribed); proficiency (% volume taken during the first 5 min/total volume prescribed); and transfer rate (mL/min).

### 2.4. Statistical analysis

Statistical analyses were performed using GraphPad Prism version 10.3.1 for Mac, GraphPad Software, Boston, Massachusetts USA, www.graphpad.com. A Shapiro-Wilk Test of normal distribution was used to determine normality prior to data analysis. Results of the Shapiro-Wilk Test found that most OFS variables (transfer volume, proficiency, and transfer rate) were not normally distributed and therefore the Friedman test, a non-parametric repeated measures test, was used to examine the effect of age on these outcome measures. OFS initial volume was normally distributed; therefore, a repeated measures (RM) ANOVA was used for this outcome variable. Post-hoc pairwise comparisons were conducted to examine differences between timepoints. Tukey's HSD was used for the RM ANOVA, and Dunn's test (Bonferroni-adjusted) was used for the significant Friedman test. An alpha level of 0.05 was used for all analyses to determine statistical significance.

## 3. Results

**Fig. 1.**
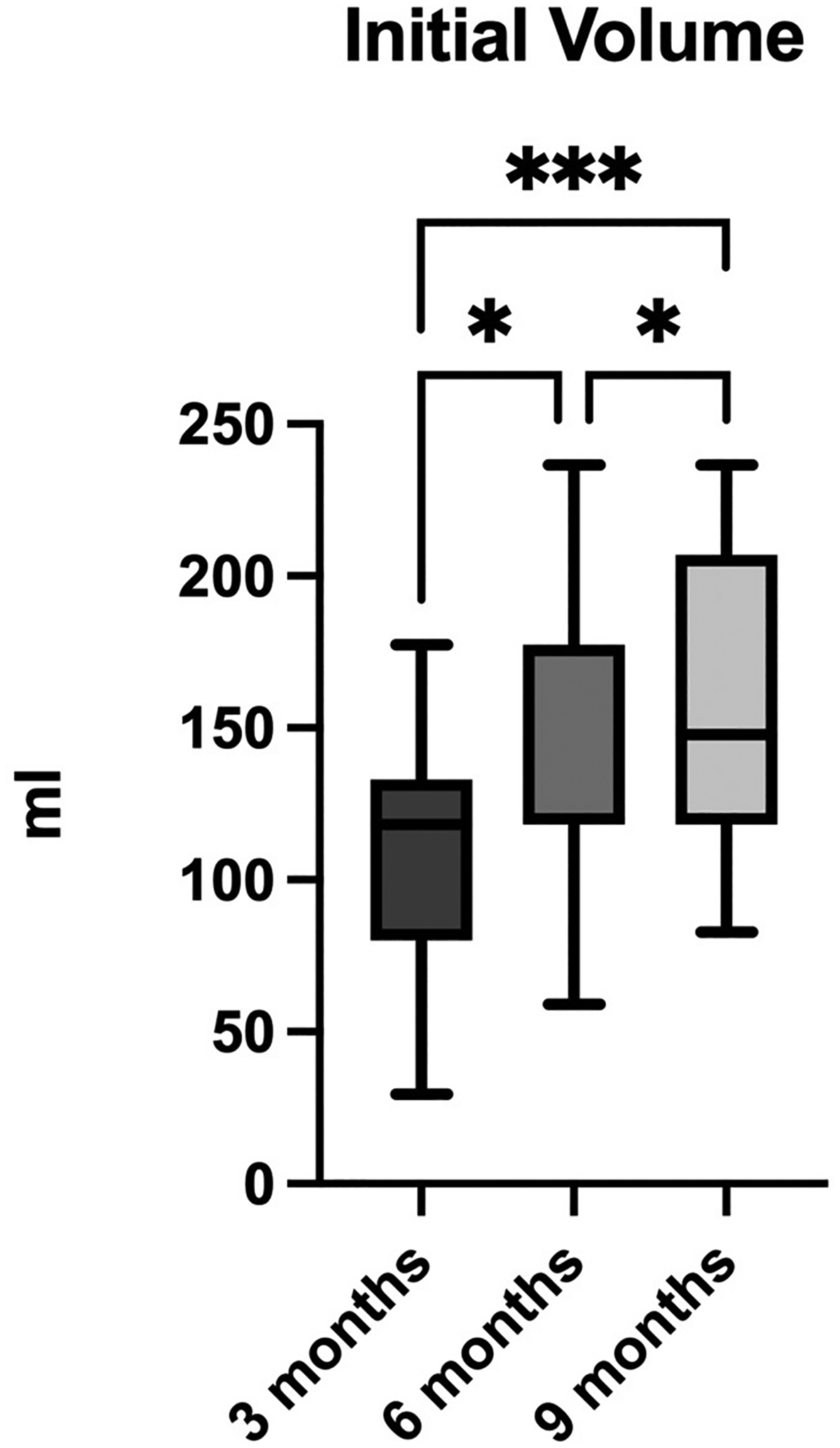
Initial volume offered by age.

RM ANOVA revealed a significant effect of age on initial bottle volume offered, *F*(1.43, 37.0) = 13.03, *p* = 0.0002, *η*^2^ = 0.33. Post-hoc comparisons showed that initial volume increased significantly across ages. Infants were offered less milk at 3 months (*M* = 110.0 mL, *SD* = 36.92) compared to 6 months (*M* = 141.4 mL, *SD* = 45.89), *q* = 4.36, *p* = 0.01 and 9 months (*M* = 156.8 mL, *SD* = 48.71), *q* = 5.93, *p* < 0.001. Initial volume at 9 months was also significantly higher than at 6 months, *p* = 0.03. See Fig. 1.

**Fig. 2.**
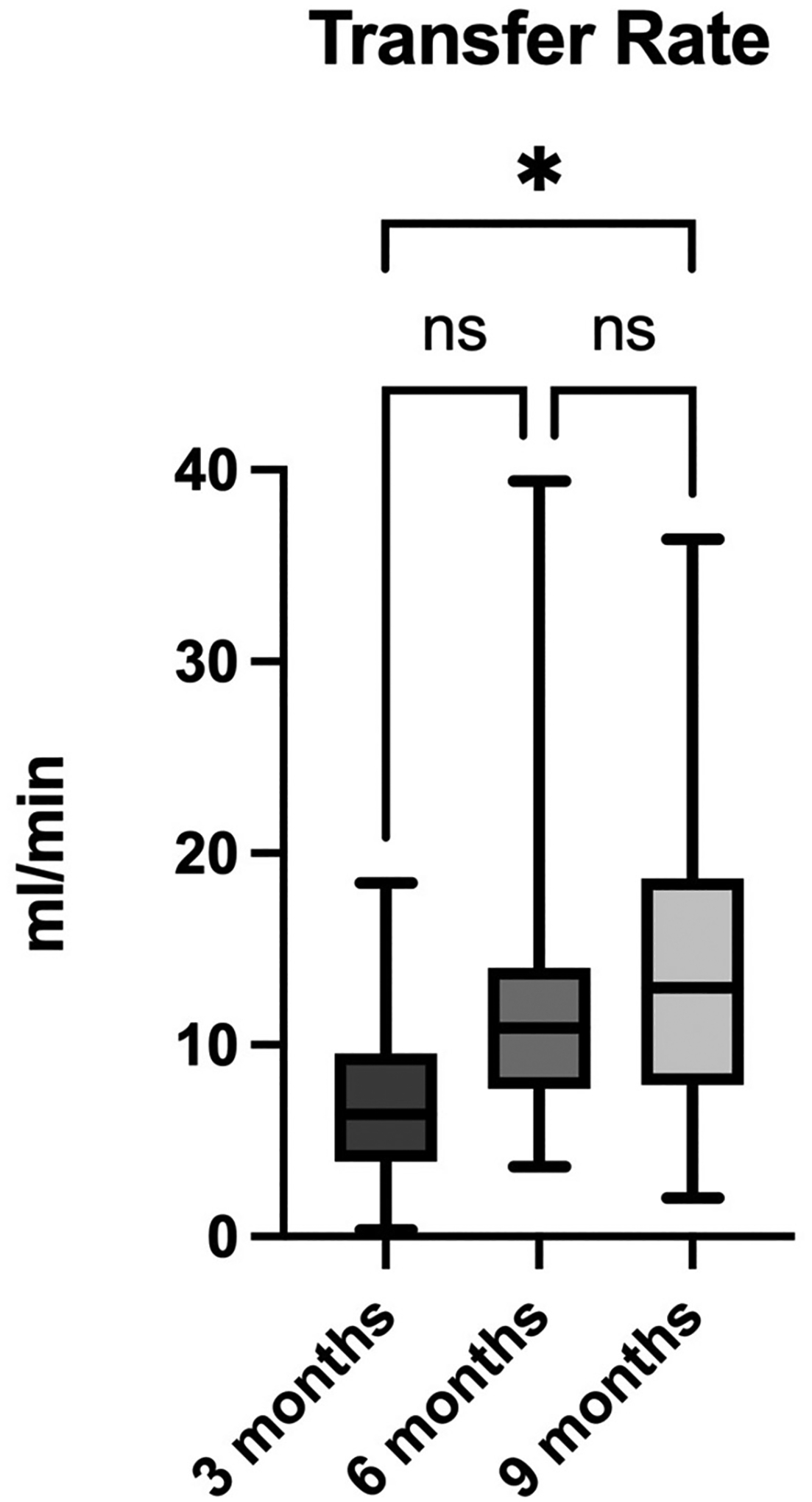
Transfer rate by age.

Friedman test results revealed a significant effect of age on OFS transfer rate, χ^2^(2) = 8.07, *p* = 0.02. Post-hoc pairwise comparisons indicated that transfer rate was significantly higher at 9 months (median = 13.00, range = 34.39) compared to 3 months (median = 6.40, range = 18.11), *Z* = 2.59, *p* = 0.03. No significant differences were observed between 3 and 6 months (median = 10.89, range = 35.79), *Z* = 2.31, *p* = 0.06 or between 6 and 9 months, *Z* = 0.27, *p* > 0.99. There were no significant effects of age on OFS proficiency, χ^2^(2) = 0.55, *p* = 0.76 or overall transfer volume, χ^2^(2) = 0.69, *p* = 0.71. See Fig. 2.

## 4. Discussion

This is the first study to analyze bottle feeding outcomes using the OFS longitudinally across the first year of life among full-term infants. Consistent with expectations, we observed a significant increase in offered milk volume as infants aged. The volume offered at 3 months was lower than at both 6 and 9 months, with a significant difference also observed between the 6- and 9-month timepoints. These findings suggest that caregivers appropriately adjust feeding volumes based on infant developmental needs and stomach capacity but maintain consistent feeding dynamics. The increased initial offering reflects caregivers' learned understanding of their infant's growing nutritional requirements, while the stable percent consumed indicates caregivers become skilled at estimating appropriate amounts regardless of age. While this was not surprising, it was important to measure as it may influence the milk transfer rate and efficiency metrics.

We found that infants displayed a significant increase in their bottle-feeding milk transfer rate between the 3- and 9-month timepoints, suggesting a developmental progression in feeding efficiency. Infants consumed more milk per minute as they matured, aligning with previous research that indicates increased oral motor control and feeding efficiency with age [13,14]. Notably, we found wide variability in initial volume of milk offer and transfer rate, aligning with prior research [12]. Despite faster feeding rates, infants maintain consistent intake patterns, suggesting a natural self-regulation mechanism. This finding warrants further exploration with a larger cohort of both full-term infants and for whom oral feeding is limited in early life.

One of the most interesting findings is that infants demonstrate remarkable consistency in feeding proficiency throughout the first year of life. Notably, no significant changes in overall feeding proficiency or transfer volume were observed at any timepoint, suggesting that factors beyond developmental maturation, such as accumulated feeding experience or individual variability, may play important roles in shaping feeding outcomes. The stability of percent volume taken and consumption within the first 5 min across different ages indicates that infant satiety cues and self regulation mechanisms remain relatively constant despite ongoing developmental changes. This finding suggests that infants may possess an inherent capacity to regulate their intake that persists even as their feeding capacity and efficiency mature, highlighting the importance of both biological and experiential factors in feeding development.

### 4.1. Clinical implications

In summary, while feeding efficiency tends to improve with age, individual variability and other developmental factors may influence feeding outcomes. This highlights the need for a personalized approach to feeding therapy, taking into account each infant's unique developmental trajectory and environmental influences, such as caregiver feeding practices and exposure to bottle-feeding.

The OFS scale serves as a quantifiable measure for feeding, supporting understanding of the proficiency and endurance of an individual infant. Deviations and limitations of total volume intake, specifically stable and proportional volume intake, may be an early indicator of feeding difficulties. The five-minute intake trial outlined in the OFS may serve as a screening tool for rapid in-person or virtual clinical evaluation of bottle-feeding. Establishing norms for bottle feeding across the first year of life can support early identification of infants with oral-motor delays, ensuring more timely intervention. This information may inform a holistic assessment of the infants' overall feeding skills and risk for demonstrating related difficulties.

### 4.2. Limitations and future directions

This study yields important findings regarding oral feeding development; however, several limitations deserve attention. First, the sample in this study was small and homogenous, representing mainly Caucasian infants from the greater Boston area. The parents who participated were largely well-educated with high socioeconomic status. Therefore, we cannot generalize these findings to the larger population and do not know if regional or cultural bottle-feeding practices may sway data. To preserve ecological validity, caregivers followed their usual bottle-feeding routines without standardizing bottle type, nipple flow, liquid viscosity, or feeding position. Consequently, while the study captured authentic feeding practices, experimental control was limited for within- and between-subject comparisons across trials. Future research is needed with larger samples across multiple regions with multiple data points during the day that may provide more insight into the stability of feeding.

## 5. Conclusions

This longitudinal study of bottle feeding across the first year of life provides insights into caregiver practices and infant maturation of bottle feeding. Results suggest that while the mechanics of feeding (speed, volume) change with development, fundamental feeding behavior and appetite regulation remain stable. Further studies of the OFS scale across the first year of development, across patient populations and with larger sample sizes, are needed.

## Figures and Tables

**Table 1:** Infant demographics information.

	3 months	6 months	9 months
Age at assessment in months (SD)	3.11 (0.38)	6.08 (0.29)	9.14 (0.32)
Sex (% male)	16 (59%)		
Birthweight in pounds (SD)	7.52 (0.94)		
Gestational age at birth in weeks (SD)	39.38 (1.21)		

Note: two participants had missing data for birthweight and gestational age at birth.

**Table 2:** Caregiver demographic information.

Maternal education level	
College degree	5 (18.52%)
Graduate degree	16 (59.26%)
Doctorate degree	6 (22.22%)
Race and ethnicity	
White	26 (96.30%)
Multiple races	1 (3.70%)
Hispanic or Latino	0 (0%)
Not Hispanic or Latino	27 (100%)
